# A Handheld Tool for the Rapid Morphological Identification of Mosquito Species (VectorCam) for Community-Based Malaria Vector Surveillance: Summative Usability Study

**DOI:** 10.2196/56605

**Published:** 2024-08-16

**Authors:** Saisamhitha Dasari, Bhavya Gopinath, Carter James Gaulke, Sunny Mahendra Patel, Khalil K Merali, Aravind Sunil Kumar, Soumyadipta Acharya

**Affiliations:** 1 Johns Hopkins University Center for Bioengineering Innovation and Design Baltimore, MD United States

**Keywords:** malaria, vector surveillance, usability study testing, usability study, usability studies, usability, usable, usableness, usefulness, utility, convolution neural networks, artificial intelligence, AI, human factors design, human factors, mHealth, mobile health, app, apps, digital health, digital technology, digital intervention, digital interventions, smartphone, smartphones, mobile phone

## Abstract

**Background:**

Malaria impacts nearly 250 million individuals annually. Specifically, Uganda has one of the highest burdens, with 13 million cases and nearly 20,000 deaths. Controlling the spread of malaria relies on vector surveillance, a system where collected mosquitos are analyzed for vector species’ density in rural areas to plan interventions accordingly. However, this relies on trained entomologists known as vector control officers (VCOs) who identify species via microscopy. The global shortage of entomologists and this time-intensive process cause significant reporting delays. VectorCam is a low-cost artificial intelligence–based tool that identifies a mosquito’s species, sex, and abdomen status with a picture and sends these results electronically from surveillance sites to decision makers, thereby deskilling the process to village health teams (VHTs).

**Objective:**

This study evaluates the usability of the VectorCam system among VHTs by assessing its efficiency, effectiveness, and satisfaction.

**Methods:**

The VectorCam system has imaging hardware and a phone app designed to identify mosquito species. Two users are needed: (1) an imager to capture images of mosquitos using the app and (2) a loader to load and unload mosquitos from the hardware. Critical success tasks for both roles were identified, which VCOs used to train and certify VHTs. In the first testing phase (phase 1), a VCO and a VHT were paired to assume the role of an imager or a loader. Afterward, they swapped. In phase 2, two VHTs were paired, mimicking real use. The time taken to image each mosquito, critical errors, and System Usability Scale (SUS) scores were recorded for each participant.

**Results:**

Overall, 14 male and 6 female VHT members aged 20 to 70 years were recruited, of which 12 (60%) participants had smartphone use experience. The average throughput values for phases 1 and 2 for the imager were 70 (SD 30.3) seconds and 56.1 (SD 22.9) seconds per mosquito, respectively, indicating a decrease in the length of time for imaging a tray of mosquitos. The loader’s average throughput values for phases 1 and 2 were 50.0 and 55.7 seconds per mosquito, respectively, indicating a slight increase in time. In terms of effectiveness, the imager had 8% (6/80) critical errors and the loader had 13% (10/80) critical errors in phase 1. In phase 2, the imager (for VHT pairs) had 14% (11/80) critical errors and the loader (for VHT pairs) had 12% (19/160) critical errors. The average SUS score of the system was 70.25, indicating positive usability. A Kruskal-Wallis analysis demonstrated no significant difference in SUS (*H* value) scores between genders or users with and without smartphone use experience.

**Conclusions:**

VectorCam is a usable system for deskilling the in-field identification of mosquito specimens in rural Uganda. Upcoming design updates will address the concerns of users and observers.

## Introduction

### Background

Malaria infects an estimated 249 million individuals annually, causing >600,000 deaths worldwide [1]. The global incidence of malaria has increased since the COVID-19 pandemic, with 13 million more cases and 63,000 more deaths [2]. According to the World Health Organization, regions in Sub-Saharan Africa are particularly susceptible to malarial infection, accounting for >95% of global malaria cases and deaths; children aged <5 years account for nearly 80% of malaria deaths in the region [3]. Specifically, Uganda has one of the highest global burdens of malaria cases, costing the country US $500 million annually [4]. In 2021, the World Health Organization estimated that there were 13 million malaria cases and nearly 20,000 malaria deaths in Uganda [4].

Currently, efforts to eliminate malaria rely on monitoring vector species composition, abundance, distribution, and behavior across different transmission geographies. Vector control strategies, such as distributing long-lasting insecticidal nets and performing indoor residual spraying of insecticide in high transmission regions, have proven to be highly effective in preventing infection [5]. However, mosquito vectors vary in their biting patterns (some may bite outdoors more frequently, and others may bite indoors more frequently) and subsequently necessitate different vector control strategies. Therefore, the Ministries of Health and their National Malaria Control Programs in almost all African countries have emphasized the need for vector surveillance, a system that allows for the collection of vector species prevalence and density data to target vector control interventions and resource allocation decisions based on the mosquito’s species-specific strategies [6]. Routine vector surveillance strategies begin with mosquito collection, where mosquito specimens are collected at sentinel sites using various vector collection methods, including Centers for Disease Control and Prevention light traps, pyrethrum spray catches, and human landing catches [7]. These specimens, collected daily, are then transported to a central laboratory. There, they are morphologically identified through microscopic examination by individuals highly trained in entomology and vector surveillance, known as vector control officers (VCOs), for their species, sex, and abdominal status. A subset of the specimens is then sent for molecular analysis through polymerase chain reactions (PCRs) and DNA sequencing when resources are available. This subset is treated as a gold standard for further confirmation and quality assurance of the species identified through vector surveillance [8]. Unfortunately, the global shortage of entomologists hinders large-scale surveillance efforts, especially in areas with a high burden of malaria, where vector surveillance is needed the most [9,10]. As a result, the sites where specimens are collected and analyzed are sparsely distributed across a target region and treated as a representation of the entire country. This causes inaccuracies in the interventions deployed, which is further worsened given the time lag between the capturing of specimens and the reporting time of usable data for decision-making [11].

To address this unmet need arising from the global shortage of entomologists, our research group at the Johns Hopkins Center for Bioengineering Innovation and Design has developed VectorCam. VectorCam is a low-cost artificial intelligence (AI)–based tool that morphologically identifies a mosquito’s species, sex, and abdomen status based on a simple photograph and concurrently uploads the summary data to a central electronic dashboard, thereby deskilling the identification and reporting process. Such a tool helps share the efforts of trained entomologists with rural community health workers, termed village health teams (VHTs) in Uganda, thereby enabling the implementation of widespread vector surveillance programs and driving better-informed, data-driven malaria intervention decisions in a cost-effective manner.

### The VectorCam System: An Overview

VectorCam enables the accurate morphological identification of mosquito species using 3 core components: specialized imaging hardware, a computer vision algorithm, and a mobile app. The hardware consists of a simple light box with a built-in 15x macro lens, paired with a basic smartphone and several mosquito trays, enabling the rapid identification of multiple mosquitos with high throughput. The VectorCam Android app is powered by an optimized version of a previously published computer vision algorithm from our laboratory, which can identify >39 species across multiple genera [12]. This adapted version of the algorithm, used in VectorCam, identifies major genera (Anopheles, Culex, Aedes, Mansonia, and nonmosquitos) and 4 different groups of species within the Anopheles genera of interest in Uganda (*Anopheles funestus* sl, *Anopheles gambiae* sl, *Anopheles stephensi*, and other Anopheles species), as Anopheles is the only malaria-carrying mosquito genera. This computationally efficient version can run locally on low-end Android phones without access to the internet, thereby allowing users to capture images of collected mosquito specimens and instantaneously obtain the classification. The app also consists of a workflow for data inputs from the user, including logistical information about the collection site, date, and other notes for data tracing. Finally, the user can capture an image of the mosquito specimen directly within the app. The algorithm then processes this image and provides the predicted species, sex, and abdominal status of the mosquito.

The imaging hardware and software user interface and user experience have been cocreated through a multiyear human-centered design approach involving inputs from >50 VHT members and VCOs from Uganda, Zambia, and Ghana. Multiple formative usability studies have been performed with nearly 40 VHTs and 10 VCOs in Uganda to finalize the hardware and app design, as well as essential performance criteria. This study serves as a summative usability assessment of a 2-person task-shifted vector identification strategy using the VectorCam app. This system was designed to fit easily into the current vector surveillance practices. For quality assurance purposes, careful consideration was taken to ensure that each mosquito was uniquely tagged for verification and molecular and PCR identification, as needed. The results of the study will inform future design iterations of the VectorCam system for field deployment in vector surveillance programs.

### VectorCam Hardware and Software Details

**Figure 1:**
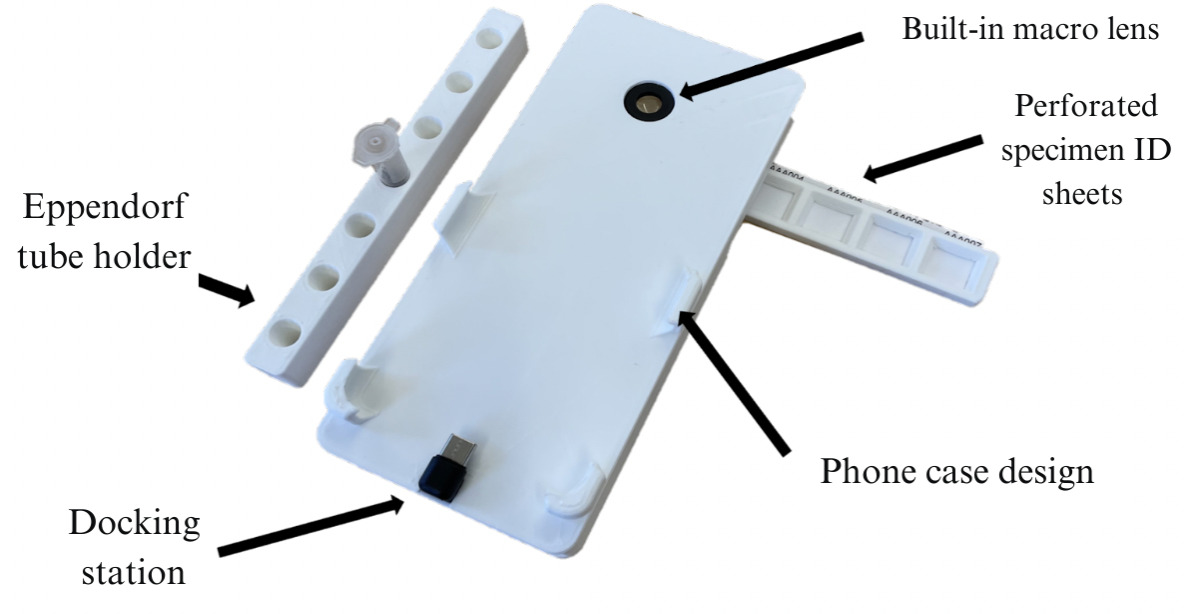
The hardware components of the VectorCam system include the light box with a built-in 15x macro lens, a phone case design, and a docking station. Hardware also includes an Eppendorf tube holder and mosquito trays and perforated specimen ID sheets to adequately pack and store these mosquitos for molecular identification after imaging.

VectorCam’s hardware consists of a light box with a 15x macro lens attached to it. The top side of this light box comprises of a phone case, where the phone slides on, plugging into the docked charger at the bottom end of this light box (Figure 1). The charger is connected to circuitry on the interior of the box, which turns on the LEDs as soon as the phone is docked, allowing uniform lighting between each specimen. The phone’s camera is then aligned with the 15x macro lens, allowing uniform magnification. The box itself also has storage compartments for 2 mosquito trays on which mosquitos are placed for ease of imaging, as well as an associated Eppendorf tube holder to hold mosquitos with their unique tag after they have been identified on the app. Within the VectorCam workflow, each mosquito must not touch one another, as this can impact DNA contamination. Each mosquito tray has a slit where a disposable piece of paper can be inserted to limit any DNA contamination. Furthermore, these slits of paper have preprinted and perforated unique specimen ID tags attached for each well. Therefore, when storing these mosquitos for PCR identification, the user can simply tear off the specimen ID and place it as well as the corresponding mosquito specimen in an associated Eppendorf tube. The Eppendorf tube holder allows for ease of packing and labeling these mosquitos for subsequent molecular identification and maintaining its traceability to the identification generated by the VectorCam system.

PCR identification will be facilitated after imaging the collected samples with VectorCam to grow the continued database of true labels of specimens. These true labels will be used to continually train the VectorCam computer vision algorithm, as it is still in development. In the ideal setting, a finalized VectorCam product will replace the need for PCRs. However, the specimen ID tear-off step will still be included in the workflow to allow for traceability and postimplementation verifications. Figure 1 shows how all these hardware components interact.

**Figure 2:**
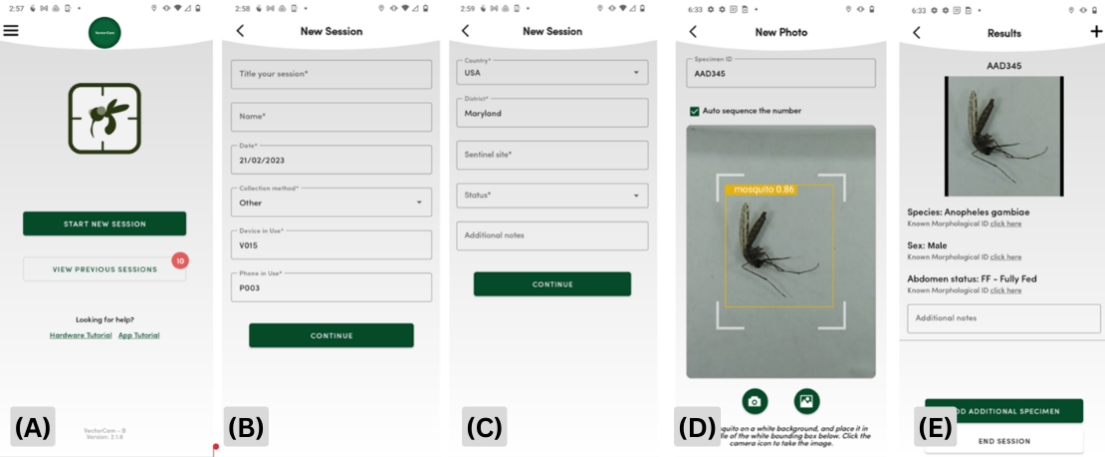
User interface screen sequences for entering background information and imaging mosquitos in order of appearance as the imager starts interacting with the app (from left to right). (A) The home screen of the VectorCam app allowing the imager to choose between starting the imaging session or viewing previous sessions already submitted in the app. (B) Under “entering background information,” the first screen allows users to enter the title of the session, the name of the user, the date of the imaging session, and information on the device and phone in use. (C) The second screen allows the user to input additional information, including country, district, and sentinel site of the current catch and imaging process as well as the status of the mosquito specimen (freshly collected specimens or desiccated). (D) The imaging screen for the imager to enter the specimen ID of the mosquito and image the mosquito using zooming and capture. (E) The results screen of the previous image of the specimen captured showing the species, sex, and abdomen status. This screen allows the imager to choose to add an additional specimen or end the imaging session.

The app itself is hosted on a Motorola G Play (2021; Motorola, Inc) smartphone. The app consists of three primary processes: (1) entering background information, (2) imaging mosquitos, and (3) submitting a session. Entering background information effectively stores all the information that entomologists usually write when generating reports for the Ministry of Health. This includes how these mosquitos were collected, the date they were collected, and the location where they were collected. Figure 2 shows details of each screen on the app. The goal of this summative usability study was to evaluate the usability of the VectorCam system among village health workers by assessing its efficiency, effectiveness, and satisfaction. We hypothesized that the VectorCam system will overall be usable with no significant differences between sex or users with and without smartphone use experience.

## Methods

### VectorCam Device Workflow

The VectorCam system involves placing collected mosquitos into the VectorCam hardware; capturing a magnified image of the mosquito using a smartphone-based mobile app for morphological identification; and storing the mosquito in an Eppendorf tube with a unique label for subsequent molecular verification, if needed.

**Figure 3:**
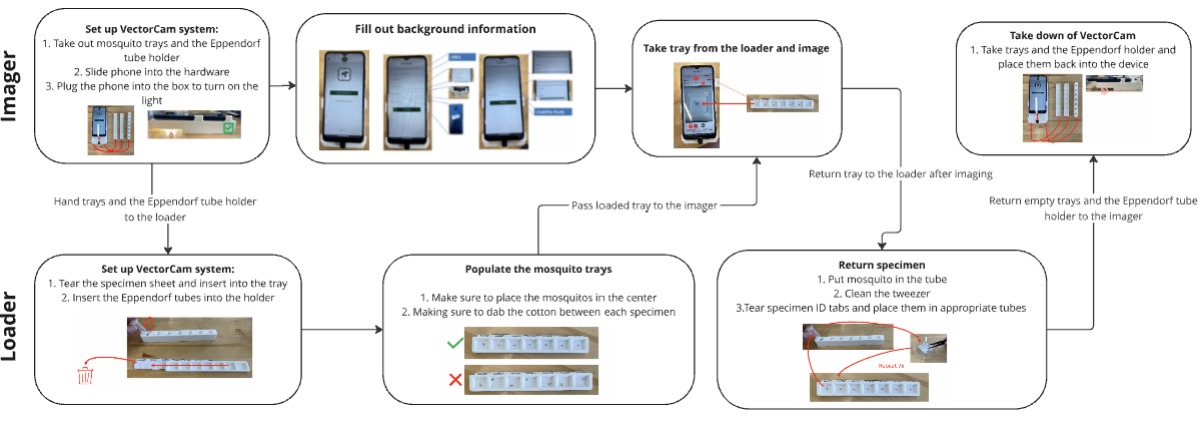
The entire workflow of the use of VectorCam, including how the imager and loader roles work together in this process. The imager primarily interacts with the specialized imaging hardware, and the loader primarily interacts with the mosquito trays and Eppendorf tube holder.

The VectorCam system was designed to be operated by a group of 2 VHT members working in collaboration by sharing the critical pieces of the workflow, whereby one person (the loader) loads specimens and another (the imager) uses the app to capture images of the mosquitos. Having a 2-person team speeds up the morphological identification process of a large batch of mosquitos. The materials used in this process are presented in Table 1, and a workflow summary of the VectorCam system is presented in Figure 3.

**Table 1:** Materials needed to operate VectorCam in a 2-user system, their purpose in the workflow, and the role of the person using the equipment.

Equipment used	Purpose of the equipment	Person using the equipment
VectorCam light box	Contains a holder for the smartphone with the VectorCam app, 15x macro lens for magnification, and a phone-powered LED light source on the underside	Imager
Motorola G Play (2021; Motorola, Inc) smartphone	Contains the VectorCam app to enter relevant background information; capture images of multiple mosquitos per session; display corresponding species, sex, and abdominal status as identified by the algorithm; and submit sessions to the cloud-based server	Imager
Plastic mosquito trays	Contains designated wells to allow for the placement of several specimens in 1 tray and for rapidly imaging all the specimens loaded onto the tray	Both imager and loader
Eppendorf tube holder	Contains designated holes for placing 7 Eppendorf tubes to facilitate easy and rapid packing of each imaged specimen into respective Eppendorf tubes along with the corresponding specimen ID label	Loader
Sheet of specimen IDs	Perforated sheets with unique specimen IDs to allow for separation and placement into the mosquito trays. The labels can be removed and placed into specific Eppendorf tubes with their associated specimens	Loader
Petri dish with cotton	Contains cotton soaked in isopropyl alcohol for periodic cleansing of the tweezers handling the specimen	Loader
Tweezers	Used for handling the mosquito specimen during tasks in the workflow	Loader
Eppendorf tubes	Used to store the specimen after the imaging process is complete	Loader

### Description of the Loader’s Role

The loader has two primary responsibilities: (1) placing mosquitos onto the mosquito trays to streamline the imaging process and (2) storing already-imaged mosquitos in individually labeled Eppendorf tubes such that the mosquitos are uniquely tagged and can be sent to a laboratory for molecular identification via PCRs or sequencing. To properly place the mosquitos onto the VectorCam hardware, the loader takes the 2 mosquito trays provided in the hardware and slides the 8 inch (length) by 1 inch (width) specimen ID sheets inside the tray. Each of these sheets has a perforated tab that appears above the tray, displaying a specimen ID for each of the wells on the tray. This allows each mosquito on the tray to have a unique label. The loader then uses tweezers to add a singular mosquito to each well, decontaminating the tweezers with isopropyl alcohol–soaked cotton before handling each mosquito to prevent DNA cross-contamination. With this sterilization, the mosquitos can be sent for molecular identification via PCRs for quality control. Once the tray is filled, it is handed off to the imager.

Once the tray is imaged, the imager passes the mosquito tray back to the loader. At this point, the loader works to unload and store each mosquito and its associated specimen ID. The loader first adds a mosquito to an Eppendorf tube that can be held on the Eppendorf tube holder provided. This is done with tweezers that are sterilized with isopropyl alcohol between mosquito transfers to prevent DNA cross-contamination. Then, the specimen ID sheet is taken out, and each ID is torn off and placed in its corresponding Eppendorf tube along with the mosquito. This gives each mosquito a unique specimen ID when it is sent for molecular identification. Therefore, quality control can be established where the gold-standard assessment of each mosquito’s species, sex, and abdominal status can also be received for further analysis.

### Description of the Imager’s Role

Once the imager receives the mosquito tray filled with specimens, they attach the phone to the light box, ensuring the LEDs are turned on, and then they go through the process of imaging the mosquitos on the app. This includes entering the background information, taking a photo of each specimen, zooming in to ensure that there is enough resolution in each photograph, and finally saving the session for later uploading to our cloud-based server.

After the imager finishes imaging all mosquitos on VectorCam, they pass the tray back to the loader, who properly stores these mosquitos. Furthermore, once the entire process of imaging is finished for the day, the imager collects all mosquito trays and Eppendorf tube holders, undocks the phone, and places the materials inside the light box for storage.

### Study Design: Materials and Methods

#### Participants and Location Selection

This summative usability study was conducted with 20 participants located in 2 malaria-endemic districts in Uganda: Mayuge and Adjumani. A sample size of 20 participants was determined using a study conducted by Faulkner [13] that collected empirical data from a sample of 60 individuals with varying levels of computer experience and varying levels of knowledge on the software used in the evaluation. In the study, a sample of 20 people was able to find a minimum of 95% and an average of 98% of the problems. Therefore, a sample population of 20 users was sufficient to encompass most of the usability problems with a device and design and a manageable number for our group to recruit, given the time constraints.

Participant recruitment criteria included those who (1) were aged between 18 and 65 years, (2) had existing status as a VHT member within Mayuge or Adjumani, and (3) had no previous experience using the VectorCam app or its previous iterations. Participants were not excluded based on prior experience with smartphone app use or with vector surveillance within their communities. Sites were selected with the support of the Ugandan Ministry of Health to ensure the heterogeneity of experience with vector control programs. At the time of the study, Mayuge had no established vector control program, while Adjumani had an established vector control program, according to the Ugandan Ministry of Health. Adjumani currently has a high burden of malaria prevalence in Uganda, as it is situated in an area of high, stable transmission of malaria [12].

Participants were recruited randomly from pools of existing VHT members in each district using the inclusion criteria described. The feedback received from the recruited VHTs serves as a valuable resource for enhancing VectorCam’s functionality and its potential impact on vector control efforts in rural Uganda.

#### Separating the Study Into Phases

During phase 1 of this study, 20 VHT members were paired with 1 VCO (out of a pool of 4 VCOs), and the VHT member was evaluated on the imager and loader roles in turns, with the VCO assuming the other role. This was done to limit any impact of another newly trained VHT member on the evaluation of outcome success criteria described in the *Outcomes* section. A total of 14 mosquitos were imaged or loaded during this evaluation. After 14 mosquitos were imaged, the VCO and VHT member switched roles so that the VHT member was evaluated for performance as both the loader and imager.

Phase 2 had 2 sets of trained VCOs imaging and 10 sets of paired VHT members imaging and loading a set of 28 mosquitos. After these 28 mosquitos were imaged, the 2 VCOs or VHT members swapped roles, allowing each participant to be evaluated in both roles. VCOs were evaluated using the same outcome measures as the VHT members, and this was done to benchmark their performance and compare it to that of the VHT pairs as a secondary analysis.

### Ethical Considerations

This study was conducted with the approval of the Johns Hopkins University Institutional Review Board (00259683) and The Aid Support Organization Research Ethics Committee approval (2022-91) from the Makerere University School of Public Health. All participants were deidentified within the data. Each participant was compensated 10,000 Ugandan Shillings for their successful completion of their portion of the study.

### Outcomes

The primary outcome of interest was the usability of the VectorCam system (device and app). This was assessed using efficiency, effectiveness, and satisfaction measurements. Each outcome of interest was observed and recorded by an observer in real time.

#### Effectiveness

The effectiveness of the use of the VectorCam system was defined by the error rates caused by the failure of critical tasks of the use of VectorCam by the VHT members. For instance, phase 1 had 4 critical tasks for the imager and loader (imager: setup, imaging—entering background information, imaging—taking pictures, and takedown; loader: setup 1, setup 2, takedown 1, and takedown 2) and phase 2 had 8 critical tasks for the loader (setup 1 to 4 and takedown 1 to 4) and 4 critical tasks for the imager (setup, imaging—entering background information, imaging—taking pictures, and takedown). To calculate the error rates, the total number of failures on these critical tasks was recorded (each task had a binary pass or fail) and summed up. This was then divided by the total number of critical tasks. To evaluate this on a participant pool basis, the number of critical tasks was summed across participants and then divided by the total number of critical tasks across participants as well.

To identify these critical tasks and subtasks, each of the authors did a cognitive walk-through of the VectorCam system workflow, asking 2 key questions for each step: “Will the user know what to do at this step?” and “Will the user know that s/he did the right thing?” [14]. After a collective discussion, a comprehensive list of all the critical tasks as well as the subtasks in each of these critical tasks was finalized. The critical tasks included (1) hardware setup, (2) populating regional background information into the app, (3) imaging of the specimens, and (4) takedown of the hardware. A success criterion was then determined for each task, and a binary pass or fail metric was established. Any failure of the success criteria contributed to the total failure of the larger critical task and the error rate. Table 2 includes lists of the subtasks performed by both the imager and loader and their success criteria during each of the 4 critical tasks.

**Table 2:** From the cognitive walk-through analysis performed by the authors of this paper, different subtasks for the process of using VectorCam for both the imager and loader roles were identified. Corresponding success criteria for both the imager’s and loader’s subtasks were identified during the setup of VectorCam. The subtasks and success criteria were identified for each main task of VectorCam: setup of the system, entering background information, taking pictures, and the takedown of the device.

Main task, role, and subtasks				Success criteria
**Setup of VectorCam**				
	**Imager**			
		Take out mosquito trays 1 and 2 and the Eppendorf tube holder and pass them to person 2.	All mosquito trays and tubes are taken out.	
		Slide the phone into the 3D-printed hardware.	The phone is slid into the 3D-printed box.	
		Place the phone into the box.	The phone is plugged into the box, and LEDs light up.	
	**Loader**			
		Write specimen IDs on a sheet of paper.	All and accurate specimen IDs are written on the paper.	
		Place the paper in the mosquito tray and Eppendorf tubes in the tube holder and puncture the Eppendorf tube.	Paper is placed in the mosquito tray, and tubes are in the Eppendorf tube holder. Eppendorf tubes are properly punctured.	
		Populate the mosquito tray with 7 mosquitos (1 in each box).	All 7 mosquitos are populated into the tray.	
**Imaging process: entering background information**				
	**Imager**			
		Create a new session on the VectorCam app and enter all the background information.	A new session is created on the VectorCam app with the correct background information and title of the session.	
		Enter specimen ID and click “auto-populate,” if sequential.	Specimen ID is entered as stated on the mosquito tray sheet.	
	**Loader**			
		Ensure mosquitos are in the center of mosquito tray 1.	All mosquitos are centered in each well in mosquito tray 1.	
		Pass mosquito tray 1 to the imager.	Mosquito tray 1 is passed to the imager.	
**Imaging process: taking pictures**				
	**Imager**			
		Pinch zoom on the mosquito (pinch diagonally across the screen).	Once the mosquito is in the camera screen, it is pinch zoomed such that the entire mosquito is in frame.	
		Take an image by clicking the camera icon and select analyze.	The camera icon is selected, and the analyze option is selected.	
		Add an additional specimen and move the mosquito tray.	An additional specimen is added, and the previous specimen is not overwritten.	
		Once 7 mosquitos have been imaged, pass mosquito tray 1 to the loader and take mosquito tray 2 from the loader.	The mosquito tray is passed to the loader.	
		Add an additional specimen and place mosquito tray 2 under the camera so that the mosquito is seen.	An additional specimen from location 1 is added.	
	**Loader**			
		Place the paper in mosquito tray 2.	The paper is placed in the mosquito tray, with specimen IDs visible.	
		Populate mosquito tray 2 with 7 mosquitos.	Mosquito tray 2 is populated with 7 mosquitos from the Eppendorf tube holder.	
		Ensure mosquitos are in the center of mosquito tray 2.	All mosquitos are centered in each well in mosquito tray 2.	
		Pass mosquito tray 2 to the imager.	Mosquito tray 2 is passed to the imager.	
		Move each mosquito from tray 1 to the respective Eppendorf tube. Place the specimen ID into the tube.	All mosquitos are moved from tray 2 to the correct Eppendorf tube with the correct specimen ID.	
		Replace the paper in mosquito tray 1 with a new sheet with the next specimen ID.	A new sheet of paper is placed in mosquito tray 1 with the correct specimen ID.	
**Takedown of the device**				
	**Imager**			
		Click “submit session” and either upload the session or save the session to upload later.	“Submit session” is selected, and the session is uploaded.	
		Unplug the phone from the device and turn off the phone.	The phone is unplugged and turned off.	
		Place trays and the Eppendorf tube holder in the VectorCam hardware.	All trays and the tube holder are placed into the VectorCam hardware.	
	**Loader**			
		Move each mosquito from tray 2 to the respective Eppendorf tube. Place the specimen ID into the tube.	All mosquitos are moved from tray 2 to the correct Eppendorf tube with the correct specimen ID.	
		Remove any remaining paper from all mosquito trays.	All pieces of paper are removed from mosquito trays.	
		Hand mosquito trays and the Eppendorf tube holder to the imager.	Trays and the Eppendorf tube holder are handed back to the imager.	

Next, to determine potential user errors in VectorCam’s workflow tasks, we performed a heuristic analysis to inspect the VectorCam’s interface and identify initial usability problems using the 10 usability heuristics proposed by Nielsen and Molich [15,16], which are listed in Textbox 1. Each of the 4 authors judged the product interface separately and created a unique list of usability problems based on the usability heuristics, thereby generating a comprehensive list of user errors. After a collective discussion, a list of user errors was finalized for each subtask. These potential errors, in addition to additional errors found with VHT members during this usability study, were used to further inform future iterations of VectorCam, if needed.

**The 10 usability heuristics proposed by Nielsen and Molich [15,16] were used to analyze the VectorCam system’s user interface to determine possible user errors and inefficiencies for future design considerations and changes.**

1. Visibility of system status
2. Match between the system and the real world
3. User control and freedom
4. Consistency and standards
5. Error prevention
6. Recognitions rather than recall
7. Flexibility and efficiency of use
8. Aesthetic and minimalistic design
9. Help users recognize, diagnose, and recover from errors
10. Help and documentation

#### Efficiency: Phase 1

Efficiency in phase 1 was defined by how much time in minutes it took for each VHT member to both image and load 14 mosquitos while paired with a VCO doing the other role. Therefore, each VHT member performed one role with the VCO and then switched so that they were also evaluated in the other role. During phase 1, the VCO was included to limit any variation in the results due to the impact of being paired with another newly trained participant.

#### Efficiency: Phase 2

Comparatively, in phase 2, a “real-world” implementation of VectorCam was tested, where 2 newly trained VHTs were paired together to image a total of 28 mosquitos, switching positions afterward so that they can both be evaluated in both the loader and imager roles. Evaluators studied efficiency by timing how long the VHT member took to load or image the 28 mosquitos, depending on their role. Therefore, in phase 2, the impact of being paired with another newly trained participant was studied. The total length of time in minutes was measured, and the average length of time to image each mosquito was calculated.

Concurrently, 2 pairs of trained VCOs worked in the 2-person system as an imager and a loader to image 28 mosquitos in 4 trays each to benchmark the performance of the VHT pairs. Because these VCOs were trained on the system for multiple months, comparing the VCOs’ average length of time to load or image each mosquito with that of the VHT pairs illustrated the impact of long-term training and exposure to VectorCam on efficiency.

#### Satisfaction

Satisfaction was defined using the System Usability Scale (SUS), a robust and validated criterion used to measure end-user usability of the product [17]. The items constituting the SUS score are listed in Textbox 2.

**The System Usability Scale was administered to each participant. Each item was answered on a scale of 1 (strongly disagree) to 5 (strongly agree). Items 2, 4, 6, 8, and 10 are those for which a lower numerical score is desired, and items 1, 3, 5, 7, and 9 are those for which a higher numerical score is desired. Each of these items was asked verbally by a translator.**

1. I think that I would like to use this system frequently.
2. I found the system unnecessarily complex.
3. I thought the system was easy to use.
4. I think that I would need the support of a technical person to be able to use this system.
5. I found the various functions in this system were well-integrated.
6. I thought there was too much inconsistency in the system.
7. I would imagine that most people would learn to use this system very quickly.
8. I found the system very cumbersome to use.
9. I felt very confident using the system.
10. I needed to learn a lot of things before I could get going with this system.

The generated score (out of 100) from the SUS was used to evaluate user satisfaction. Because the SUS score can be similarly structured to that of a 100-point academic scale, 90 to 100 points was an A, 80 to 89 points was a B, and so on, with the standard average score being 68 [15]. A score >68 was passed for a positive usability assessment, and a score >80 indicated excellent usability. To further understand the functionality of the devices and the rationale for the scores given, open-ended interviews with participants were also conducted.

Secondary outcomes of interest included the following: (1) the impact of age and previous smartphone use on satisfaction and (2) the thematic clustering of voiced barriers to usability from open-ended interviews following the completion of study procedures.

### Study Execution Procedures: Materials and Methods

#### Overview

Study instructions and a user ID were verbally provided to each participant. After finishing the evaluation stage of this procedure, SUS and demographic questionnaires were administered verbally to local interpreters.

The study was conducted in five segments, namely (1) initial training and certification; (2) phase 1: initial testing with a VCO partner; (3) phase 2: testing in VHT pairs; (4) SUS and demographic questionnaires; and (5) planned statistical analysis.

#### Initial Training and Certification

All VHT members recruited were onboarded with a standardized training protocol (Multimedia Appendix 1) delivered by expert VCOs familiar with the VectorCam hardware, software, and vector surveillance workflow. VCOs trained VHT pairs to use the VectorCam hardware and software. During the training protocol, any questions asked by participants were addressed as part of the initial training session. After initial training was completed, the VCO trainers formally assessed the ability of VHT participants to use the VectorCam system in the roles of an imager and a loader. A standardized certification checklist was used to determine whether each member of the VHT pair could independently use the VectorCam system. A detailed list of certification criteria is presented in Multimedia Appendix 2. If a participant did not meet certification requirements, VCO trainers clarified and corrected errors before another attempt at certification was undertaken. The participants were then brought to the study session 24 hours after training certification to allow for sufficient natural decay of memory.

#### Phases 1 and 2: Evaluating Performance

Throughout the study sessions in both phases, an observer focused on either the imager or the loader by noting the time taken to image each tray of mosquitos, the number of errors in critical tasks in the workflow, and any observational artifacts worth noting about usability. In phase 1, one observer focused on the VHT member in the VHT and VCO pair, evaluating them in both the loader and imager roles. In phase 2, two observers were placed per pair, with 1 observer matched with 1 VHT member (so that the observer could evaluate 1 VHT member in both the imager or loader roles).

#### SUS and Demographic Questionnaires

The SUS was administered once to each participant in the study after the completion of both the roles, an imager and a loader. Each participant was administered this orally, with the question and the scoring scale being described. Their answers were then recorded, along with any qualitative feedback they had with each answer.

In this usability study, a variety of ages, genders, and technological savviness were encompassed in the participant population. A total of 8 participants were chosen for the study from Mayuge and 12 from Adjumani. Each participant completed an orally administered questionnaire, which collected data on their age, gender, experience using a smartphone, experience with vector surveillance processes, and the length of time served as a VHT member. Besides the question on the number of years of VHT experience, all other questions were answered using a standard yes or no format for ease of standardization. This questionnaire is presented in Multimedia Appendix 3.

#### Planned Statistical Analysis

A Kruskal-Wallis 1-way analysis (α=.05) was performed to test for differences in SUS scores based on (1) the gender of the participant and (2) the participant’s experience with a smartphone. This test was selected as it would prove to be more stable in case of any outliers in the samples.

After using VectorCam, each participant was questioned individually by both a translator and the study designers (authors BG, SD, and KKM). There was no time limit set for answering questions, and all users were also able to provide qualitative comments at the end of the survey.

## Results

### Participant Demographics

Of the 20 participants, 14 (70%) were male and 6 (30%) were female, with a mean age of 36 (SD 9.8) years. A total of 8 (40%) participants were aged 30 to 40 years, 6 (30%) participants were aged 20 to 30 years, 4 (20%) participants were aged 40 to 50 years, and only 2 (10%) participants were aged >50 years. A total of 12 (60%) participants had experience using a smartphone, and 4 (20%) participants had experience with vector control strategies (notably, all these participants were from Adjumani).

### Phase 1: Partner With VCO

#### Efficiency

Efficiency was defined as the time taken (in seconds) to image 14 mosquitos in phase 1. During phase 1 of the study, where each VHT member was paired with a trained VCO, each VHT member both imaged and loaded 14 mosquitos while in their 2 different roles. The average time spent imaging per mosquito for 20 VHT members was 70 (SD 30.3) seconds, and the average time spent loading per mosquito was 50 (SD 12.4) seconds.

#### Effectiveness

Effectiveness was analyzed based on the failure rates demonstrated by the VHT members when using VectorCam, which were categorized as a failure in the critical tasks outlined for the imager and the loader. During phase 1, for VHT members, there was an 8% (6/80 critical errors) error rate for the imager and a 13% (10/80 critical errors) error rate for the loader. The most frequently failed tasks for the imager and the loader were during imaging: entering the background information and setting up trays (3/20, 15% and 8/40, 20%, respectively). A larger breakdown of the error rates of both the imager and loader is provided in Tables 3 and 4, respectively.

**Table 3:** Effectiveness and error rates and number of errors for each critical task for the imager role in both phases 1 and 2, including the error rates for vector control officers (VCOs) in phase 2. Overall, 20 village health teams were evaluated in each phase, and 4 VCOs were evaluated in phase 2.

Imager	Setup, n (%)	Imaging			Takedown, n (%)
		Entering background information, n (%)	Taking pictures, n (%)		
Phase 1 (n=20)	0 (0)	3 (15)	2 (10)	1 (5)	
Phase 2 (n=20)	0 (0)	3 (15)	5 (25)	3 (15)	
VCO phase 2 (n=4)	0 (0)	1 (25)	1 (25)	0 (0)	

**Table 4:** Effectiveness and error rates and number of errors for each critical task for the loader role in both phases 1 and 2, including the error rates for vector control officers (VCOs) in phase 2. Overall, 20 village health teams were evaluated in each phase, and 4 VCOs were evaluated in phase 2.

Loader	Setup 1, n (%)	Setup 2, n (%)	Setup 3, n (%)	Setup 4, n (%)	Takedown 1, n (%)	Takedown 2, n (%)	Takedown 3, n (%)	Takedown 4, n (%)
Phase 1 (n=20)	3 (15)	5 (25)	—^a^	—	1 (5)	1 (5)	—	—
Phase 2 (n=20)	7 (35)	3 (15)	3 (15)	2 (10)	2 (10)	1 (5)	0 (0)	1 (5)
VCO phase 2 (n=4)	0 (0)	0 (0)	0 (0)	0 (0)	0 (0)	0 (0)	0 (0)	0 (0)

^a^Not applicable.

### Phase 2: Two VHT Participants Working Independently

#### Efficiency

**Figure 4:**
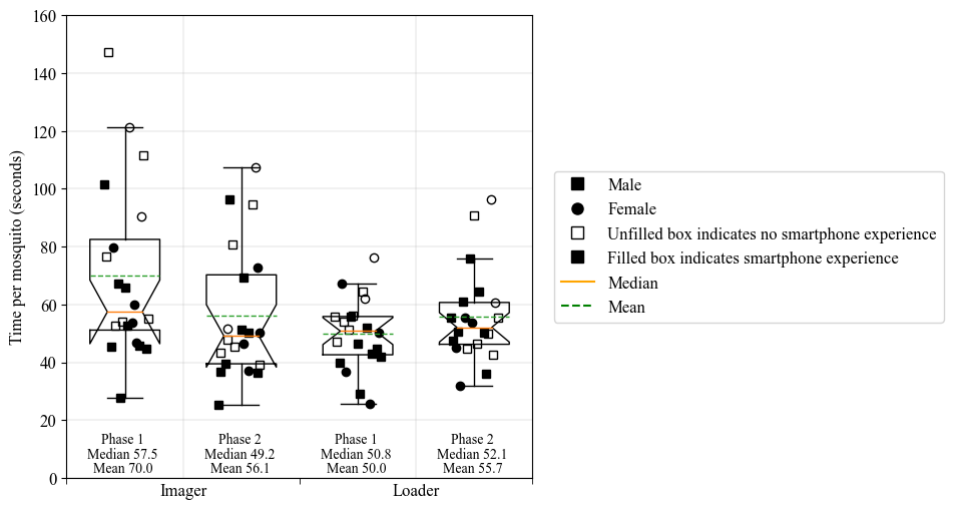
The box-and-whisker plots for efficiency metric in both phases and roles. The first 2 plots show the efficiency plots for the imager role in phase 1 and phase 2, respectively. Both the median and the mean time per mosquito decreased from phase 1 to phase 2. The third and fourth plots are the efficiency plots for the loader role in phase 1 and phase 2, respectively. Conversely, for the imager role, both the median and mean time per mosquito increased from phase 1 to phase 2.

During phase 2, a total of 48 mosquitos were imaged by the VHT member in the imager role, and the average time spent imaging per mosquito was 56.1 (SD 22.9) seconds for the VHT members. The average time spent loading per mosquito was 55.7 (SD 16.3) seconds. When phase 2 was performed by 4 VCOs with much higher training and previous smartphone experience, they were able to accomplish a throughput of 22.4 (SD 4.0) seconds per mosquito. The box-and-whisker plots for the 2 phases of efficiency and both roles are consolidated in Figure 4.

#### Effectiveness

During phase 2, there was a 14% (11/80 critical errors) error rate for the imager (for VHT pairs) and a 12% (19/160 critical errors) error rate for the loader (for VHT pairs). The most frequently failed task for the imager was imaging: taking pictures. The specific subtask that was the most erroneous was the zooming in of the specimen. For the loader, the most frequently failed task was during the setting up of trays (most notably, on the first tray, as seen in Table 4), with the most erroneous subtasks being the puncturing of the Eppendorf tubes and the transfer of the mosquito specimen to the tray. When phase 2 was performed by the 4 VCOs, the total failure rates for the imager role and loader role were 13% (2/16 critical errors) and 0% (0/32 critical errors), respectively, with “imaging: taking pictures” being the most frequently failed task for the imager role. It is important to acknowledge the smaller sample size of VCOs during this evaluation, and for a one-to-one comparison with VHT pairs, a larger sample size must be obtained.

A larger breakdown of the error rates of both the imager and loader is provided in Tables 3 and 4, respectively.

#### Comfort

**Figure 5:**
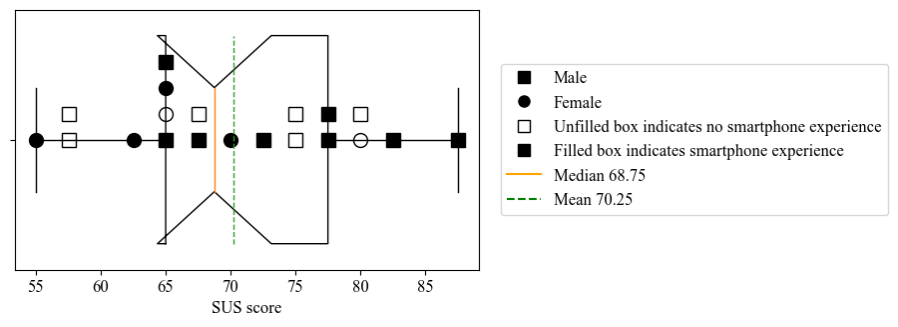
System Usability Scale (SUS) score of study participants. Each participant is stratified by sex as well as experience with a smartphone. The mean SUS score was 70.25 (SD 8.99) for all participants. Using a Kruskal-Wallis test, it was revealed that there was no significant difference in SUS scores based on smartphone experience or gender (α=.05).

At the end of the training and validation period, the SUS score and respective background information were collected. On average, the SUS score reported by participants was 70.25 (SD 8.99). The average scores across all positive and negative items were 4.48 (SD 0.72) and 2.86 (SD 0.71), respectively. The box-and-whisker plot for the distribution of the SUS scores is presented in Figure 5. The item that serves as a statistical outlier is item 10 (“I needed to learn a lot of things before I could get going with this system”).

**Figure 6:**
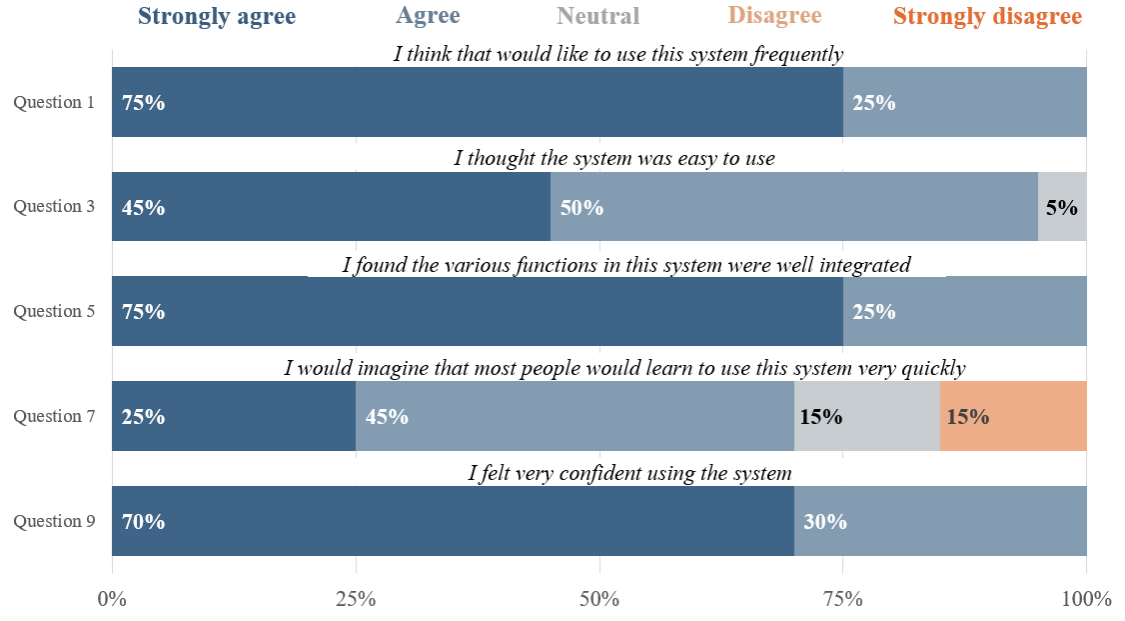
The distribution of the System Usability Scale (SUS) scores for each of the positive usability questions asked in the poststudy interview of the participants. The figure demonstrates that most users answered with a score of 4 or 5 (agree or strongly agree, respectively) for each question, as seen by the higher percentages.

**Figure 7:**
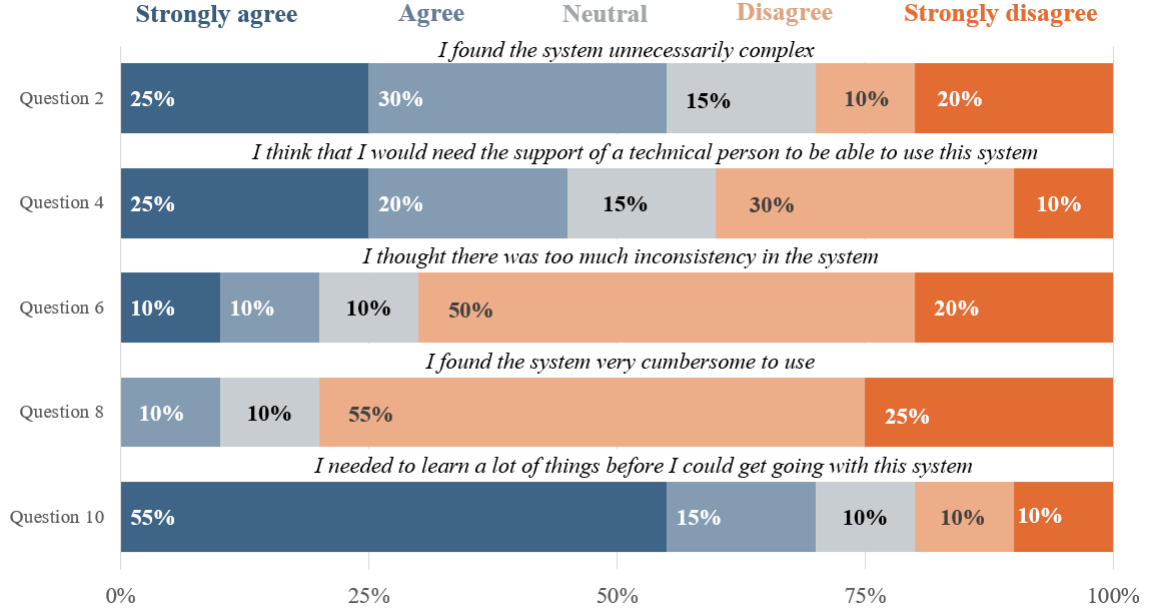
The distribution of the System Usability Scale (SUS) scores for each of the negative usability questions asked in the poststudy interview of the participants. The figure demonstrates that most users answered with a score of 1 or 2 (strongly disagree or disagree, respectively) for each question, as seen by the higher percentages. However, the last question demonstrates the opposite, with most users answering with 4 or 5 (agree or strongly agree, respectively), indicating that users felt that they needed extensive training before using the device properly.

An analysis of the satisfaction of the usability of the device was performed by looking at the difference in SUS rankings for the positive items versus the negative items in the SUS questionnaire. Figures 6 and 7 show the distribution and percentages of each SUS ranking stratified by the positive or negative categorization of the items.

### Secondary Results

#### Kruskal-Wallis Results of the Effects of Gender and Smartphone Experience on Satisfaction

The results of this usability study furthermore revealed that the SUS scores did not exhibit significant differences between the participants across Adjumani and Mayuge (N=20) based on gender or prior experience with smartphones. Using a Kruskal-Wallis test with a significance level of α at .05, there was no statistically significant variance in SUS scores based on gender or prior experience with smartphones (*H* value=0.006).

#### Thematic Clustering of Participants’ Qualitative Feedback

Themes from the postsession interviews were gathered from the feedback given by users about certain qualms of the workflow or interactions in the device. Textbox 3 highlights the main themes of the concerns voiced by the users, along with participant quotes.

**Themes from qualitative comments provided by participants after administering the System Usability Scale. All participants are anonymized and are therefore listed as U1 to U20. Quotes were translated into English for the purposes of this research.**

- Need for better training to adequately develop skills needed to use VectorCam
  “The information from the trainer needs to be clear, or there should be something we can use to know we know we are doing [the imaging process] well.” [U15]
- Difficulty handling the specimens with tweezers
  “It is hard to center the specimens [on the mosquito tray tables] because they break as you use [the tweezers].” [U13]
  “The biggest challenge was the tweezers being hard to use.” [U15]
  “Putting specimen in tubes was difficult to do with forceps.” [U20]
- Difficulty and confusion when attributing a specimen ID to each mosquito
  “The Specimen ID is very confusing, and I do not understand how the numbers increment.” [U9]
  “The numbers are very confusing; it is confusing to have both letters and numbers [in the specimen ID].” [U18]
  “The names of [the mosquitos] are hard to remember.” [U20]
- Lack of understanding of the impact of the data being recorded and hesitancy in using VectorCam without knowing this impact
  “I’m not sure where the data goes in general.” [U18]
  “I want to know where the data is being sent to.” [U14]

## Discussion

### Principal Findings

This study is the first summative field usability assessment of VectorCam, an AI-based tool that automatically detects a mosquito’s species, sex, and abdomen status, thereby deskilling the identification process. This tool has been designed and manufactured with multiple iterations of input from field users, VCOs, and engineers to be low cost and usable in rural regions in Africa. The results of this study demonstrated that the VectorCam system is overall generally usable in terms of its effectiveness, efficiency, and satisfaction among 20 VHTs in 2 districts in Uganda. A Kruskal-Wallis statistical test revealed that there is no significant difference in usability between sex and end users with and without smartphone user experience.

This study had inherent limitations that shaped the scope and interpretation of its findings. Due to a lower sample size and this study being underpowered, we are unable to confidently claim statistically significant differences in accuracy and throughput based on smartphone use. Furthermore, this study was conducted in an acute short-term setting, where VHT members had limited exposure to the VectorCam device and app (on the magnitude of 6 to 8 hours) before being evaluated on their use of the system. Comparatively, in a programmatic implementation of VectorCam, each VHT member would have multiday exposure to the device before using it independently. Prior usability studies involving medical or field technologies with users who have had long-term training on the device or previous experience with similar products demonstrated higher accuracy in using the device in terms of lower error rates and higher efficiency [18]. A notable example was seen in the lower error rates of medical professionals using a pulse oximeter with a new design [18]. Therefore, results on the accuracy as well as throughput using the VectorCam system may be skewed and could perhaps be improved given a longer training period.

The usability of the platform should also be reassessed following long-term use to further study its efficacy, satisfaction, and accuracy. Comparing the results of phase 1 with phase 2 of this study, the time taken to image each mosquito decreased by an average of 13 seconds per mosquito, as the participants had increased exposure to the device. Comparatively, the time taken by VCOs to image a mosquito was nearly half of that taken by the VHT members, showing that with more training and exposure to using the device, throughput could further increase. As shown in a study evaluating case studies of accessible digital technology for education in a low- and middle-income country setting, the programmatic implementation of tools over a longer period with good transfer and retention of knowledge on how to use them had more success in implementation compared with short-term training and evaluation [19]. Notably, the time taken to identify mosquito vectors using VectorCam was still a considerable improvement over prior methods of vector surveillance, which take a few days, and the time taken to image a mosquito using VectorCam (60 seconds) matched our success criteria [20].

To further improve this throughput, key design changes can be made. The 2 critical steps that took the longest time for the imager were entering the background information on the app as well as entering the specimen ID per mosquito. This study was conducted using an app with English as the primary written language. While English is the national language of Uganda, local dialects vary heavily between regions; therefore, the app would benefit from pictographic or video-based prompts rather than written ones for generalized use or use outside of urban settings. A subgroup analysis for literacy in English was not conducted but would be important to include in further studies.

Furthermore, the outcome measures were captured by a researcher observing the participants and recording the outcome measures manually in real time. Therefore, it is possible that critical errors were missed, and efficiency times may not be as precise as they would have been if they were coded post hoc using video recordings of each participant.

The error rates of the VHT members tested in the imager and loader roles reveal that VectorCam’s usability issues need to be addressed with revisions to the software and hardware. An important aspect to consider during the analysis of these rates is the training protocol provided to the VHT members by the VCOs, as mentioned previously. Because of the acute nature of the training and testing of these users, longer exposure and use of the VectorCam system will demonstrate the extent of these failed tasks and the possible reduction of high error rates. This hypothesis can be observed by the 4% (2/48 critical errors) error rate of the tested VCOs compared with the 13% (30/240 critical errors) error rate of the VHT members during phase 2. With this information, we can speculate that with longer training, the error rate would further decrease.

The task with the highest failure rate for the imager was taking pictures on the app. The subtask within taking pictures that was most prone to error was the zooming in of the specimen during the imaging process, which was omitted or not performed thoroughly by the imagers in some cases. To address these design inefficiencies, some design recommendations include a built-in auto-zoom feature that prevents the need for the user to zoom into the specimen in addition to adding a user profile to prevent typing of the background information. The specimen ID can also be automated, as mentioned previously, through an optical character recognition feature. These key design changes would create improvements in both types of error rates noted, as well as increase the throughput by putting less burden on the user to enter these metrics.

For the loader role, the task with the highest failure rate for both phases was the setting up of the mosquito trays. The researchers observed that the transfer of the mosquito specimen to the tray and puncturing of the Eppendorf tube were the subtasks prone to error in this task. Therefore, some hardware design revisions were proposed, such as a sliding edge to eliminate the use of tweezers for transferring mosquito specimens. Another design idea is a clasping apparatus with a sharp edge attached to the Eppendorf tube holder to automatically puncture the standing tubes with a single closing motion. This could prevent any accidental punctures to the skin, which is a potential hazard with the current process using tweezers.

### Conclusions

Vector surveillance and entomological classification are the cornerstones of malaria prevention. Through this study, it has been demonstrated that VectorCam, an AI-based tool for task-shift vector surveillance, can effectively empower VHTs to conduct vector surveillance. This study has illustrated VectorCam’s usability and accessibility, showing its potential to task-shift the time-consuming and resource-intensive process of vector identification to VHTs embedded in malaria prevention strategies within their communities. Modifications to the hardware and software solutions that are currently in progress are needed to ensure optimal usability and are the current focus of ongoing efforts by our research group. Furthermore, from September 2023 to August 2024, our team is conducting a randomized controlled trial of VectorCam to evaluate the long-term use of VectorCam in the hands of VHTs over a 12-month period. Nevertheless, VectorCam serves as a promising technology that can transcend barriers in traditional vector surveillance through task-shifting malaria prevention efforts, creating a new 21st century approach to community-based malaria vector surveillance in rural Africa and beyond.

## Appendix

Multimedia Appendix 1 The VectorCam training protocol script that each vector control officer had to follow as they trained each village health team (VHT) member in the use of VectorCam. This involves an introductory script, where all parts of VectorCam are explained. Afterward, this describes the protocol that must be followed to train each VHT member in the imager and loader roles. Before completing the usability test, they must be able to perform these steps once alone, with no errors.

Multimedia Appendix 2 This checklist details the steps that the village health team (VHT) members needed to perform to demonstrate their ability to complete critical tasks independently, for both the imager and the loader roles. Only when each of these tasks were checked off by a vector control officer, the VHT members were able to participate in the usability study. This was done to ensure that all VHT members were trained to a standardized level.

Multimedia Appendix 3 This questionnaire was administered to each village health team member by a vector control officer before conducting training or any part of the usability assessment. In this questionnaire, demographic questions about their age, gender, smartphone use, and vector surveillance experience were asked.

## Abbreviations

AI: artificial intelligence
PCR: polymerase chain reaction
SUS: System Usability Scale
VCO: vector control officer
VHT: village health team
