# Supplementary material for: A Handheld Tool for the Rapid Morphological Identification of Mosquito Species (VectorCam) for Community-Based Malaria Vector Surveillance: Summative Usability Study
Source: JMIR Hum Factors. 2024 Aug 16;11:e56605. doi: 10.2196/56605 (PMC11364941; doi:10.2196/56605)
Supplement: Multimedia Appendix 1 [file humanfactors_v11i1e56605_app1.pdf]

VectorCam Training Protocol Script

Introduction Script:

Hello Everyone!
Welcome to Training for VectorCam. VectorCam is an invention that will help with making identifying mosquitos very fast and also very accurate. Today, we will be teaching you all how to use VectorCam because one day, you all will be using VectorCam yourselves. After we are done teaching you how to use it, we will give you some time to practice using it. If we see that you need help with any part, we will reteach you that step.

Once everyone is ok with using VectorCam, we will ask you to use it for imaging 56 mosquitos. These students from Johns Hopkins University will be observing you during this and recording some important things on their end.

VectorCam is a two-person system and we will teach you steps for each person's role. We will start with first explaining the different parts of the VectorCam system and then go into the steps for each role.

Hold up the white box without phone
This is the VectorCam box.
take out the trays
This has two mosquito trays and they are located here in the box with the "T" label.
take out tube holder
It also has an Eppendorf Tube holder and is located here in the box with an "E" label.

Show phone
This will also come with a phone. This phone will go onto this VectorCam box and will plug into the box to turn on the lights.

**Show where the light is on the box*.*

show app icon on phone
On this phone is the VectorCam app -. This app is where you will be doing all of the imaging. Slide up and search for the app.

Now that we know all the components of the VectorCam solution – we will now start with explaining each Person's tasks as we go through how to use VectorCam.

Set-Up of VectorCam Hardware

Person 1 (Imager)

1) Take out two mosquito trays and one Eppendorf tube holder out of the VectorCam box. They are inside the sides of the box.
2) Give the two mosquito trays and one Eppendorf tube holder to the Loader (Person2).
3) Slide the phone onto the top of the VectorCam box between the phone grips.
4) Make sure the lights under the VectorCam box turn on.

Person 2 (Loader)

1. Puncture the Eppendorf tube and place into the Eppendorf holder
2. Tear the specimen sheet along the dotted line
   a. Start with the set of labels at the at the top of the sheet
3. Place the torn specimen sheet into the mosquito trays
   a. Slide the sheet into the slot in each tray
   b. Ensure the specimen labels are visible at the top of the tray
4. Place one mosquito in each well of the tray (7 mosquitos per tray)
   a. Clean tweezer tips between mosquitos

5. Make sure the mosquitos are in the centre of each well

Imaging Process: Enter Background

Person 1 (Imager)

1) Press on “Create New Session” on the app
3) Enter the “U0XX” that is at the top of the sheet into the “Your Name” box
4) Click the date you are taking the images for “Date” box
5) Pick which way the mosquitos were caught in the “Method of Catching” box. Ask your VCO for exact answer.
6) Enter in the number on the device for “Device in Use”.
7) Enter in the number on the phone for “Phone in Use”.

8) With the first tray of mosquitos from the loader, look at the first Specimen ID. Put Specimen ID in the “Specimen ID” box.
9) Look and make sure the “Auto sequence” box is clicked

Person 2 (Loader)

1. Double check to make sure that the mosquitos are in the centre of each well
    a. If not, move them to the centre, making sure to clean the tweezer tips in between mosquitos
2. Carefully pass the tray to Person 1 (Imager)
3. Load the second tray using the same steps as above
4. Make sure the specimen labels placed in the tray follows the order from the last tray
5. Once Person 1 (Imager) finishes imaging the mosquitos in the tray they will pass this tray back to you
6. Take the mosquitos out of the tray and place them in the Eppendorf tubes, one mosquito per Eppendorf tube
    a. Clean tweezer tips between each mosquito
    b. Tear off each specimen label and place it in the matching Eppendorf tube
    c. Discard paper scraps into a rubbish bin
7. Continue loading and unloading trays until all mosquitos are imaged

Imaging Process: Taking Pictures

Person 1 (Imager)

1) Put the mosquito tray under the box and see the first mosquito in the camera
2) Zoom in on the mosquito to make it fill the white box on the camera. It is very important that you do this step, because it a big step for the app to work correctly.
3) Next, click on the Camera Icon on the screen.
4) Next, click on the “Analyze Results” button.
5) After the results come in, click the “->” arrow on the screen to add additional specimen
6) Take pictures of all 7 mosquitos in the tray.
7) After all of the 7 mosquitos are done, move the mosquito tray back to Loader (Person 2)
8) Take the new mosquito tray with new mosquitos from Loader (Person 2)
9) Take pictures of all 7 mosquitos in this new tray will all the same steps. Make sure you zoom in one mosquito.

Person 2 (Loader)

N/A

Takedown of VectorCam Device

Person 1 (Imager)

1) After all trays of mosquitos are done, click on “Submit Session”.
2) Ask your VCO if you should *Upload the Session* or *Save Session for Later*
3) Slide the phone off of the plug to turn off the lights under the box.
4) Turn off the phone by clicking the power button on the side.
5) Take any mosquito trays and the Eppendorf tube holder back from Person 2
6) Put the mosquito trays and Eppendorf tube holder back into the same spots into the Box.

Person 2 (Loader)

1. Once all mosquitos have been imaged, place the two trays back into the Box (in the lower left corner)
2. Place the Eppendorf tube holder back into the box (in the lower right corner)
3. Task completed
