# Supplementary material for: A Handheld Tool for the Rapid Morphological Identification of Mosquito Species (VectorCam) for Community-Based Malaria Vector Surveillance: Summative Usability Study
Source: JMIR Hum Factors. 2024 Aug 16;11:e56605. doi: 10.2196/56605 (PMC11364941; doi:10.2196/56605)
Supplement: Multimedia Appendix 2 [file humanfactors_v11i1e56605_app2.pdf]

Trainee ID: ___________________________________
Trainer Name: ___________________________________
Imager:

- Successfully set-up VectorCam
  - Phone on the box
  - Trays out of the box
- Successfully start session
- Successfully populate the background information
- Successfully enter the Specimen ID
- Successfully enable auto-sequence
- Successfully image a mosquito
  - Remember to pinch zoom diagonally such that the mosquito is in the white bounding box
- Successfully go to next mosquito
- Successfully pass the tray
- Successfully end session

Trainee Signature: ___________________________________ Date: ___________
Trainer Signature: ___________________________________ Date: ___________

Trainee ID: ___________________________________
Trainer Name: ___________________________________
Loader:

- Successfully receive tray and Eppendorf tube holder
- Successfully place paper in tray
- Successfully place 7 mosquitoes in the tray, one in each well
- Successfully hand tray over with correct numbering
- Successfully cleanse tweezers between each mosquito
- Successfully place 7 mosquitoes and Specimen ID in Eppendorf tubes, one in each tube
  - Place mosquito in Eppendorf *before* the Specimen ID sheet
- Successfully repeat process for tray 2

Trainee Signature: ___________________________________ Date: ___________
Trainer Signature: ___________________________________ Date: ___________
