# Supplementary material for: A Handheld Tool for the Rapid Morphological Identification of Mosquito Species (VectorCam) for Community-Based Malaria Vector Surveillance: Summative Usability Study
Source: JMIR Hum Factors. 2024 Aug 16;11:e56605. doi: 10.2196/56605 (PMC11364941; doi:10.2196/56605)
Supplement: Multimedia Appendix 3 [file humanfactors_v11i1e56605_app3.pdf]

VHT Number: **VCO Interviewing:**

1. What is your age?
2. What is your gender?
3. What is your experience using a smartphone?
4. What is your experience with vector surveillance?
5. How long have you been a community health worker?
